# Demonstrating the importance of porcine reproductive and respiratory syndrome virus papain-like protease 2 deubiquitinating activity in viral replication by structure-guided mutagenesis

**DOI:** 10.1371/journal.ppat.1011872

**Published:** 2023-12-14

**Authors:** Ben A. Bailey-Elkin, Robert C. M. Knaap, Anuradha De Silva, Ilse M. Boekhoud, Sandra Mous, Niek van Vught, Mazdak Khajehpour, Erwin van den Born, Marjolein Kikkert, Brian L. Mark

**Affiliations:** 1 Department of Microbiology, University of Manitoba, Winnipeg, Manitoba, Canada; 2 Molecular Virology Laboratory, Department of Medical Microbiology, Leiden University Center of Infectious Diseases (LU-CID), Leiden University Medical Center, Leiden, The Netherlands; 3 Department of Chemistry, University of Manitoba, Winnipeg, Manitoba, Canada; 4 MSD Animal Health, Boxmeer, The Netherlands; Washington State University, UNITED STATES

## Abstract

Deubiquitination of cellular substrates by viral proteases is a mechanism used to interfere with host cellular signaling processes, shared between members of the coronavirus- and arterivirus families. In the case of Arteriviruses, deubiquitinating and polyprotein processing activities are accomplished by the virus-encoded papain-like protease 2 (PLP2). Several studies have implicated the deubiquitinating activity of the porcine reproductive and respiratory syndrome virus (PRRSV) PLP2 in the downregulation of cellular interferon production, however to date, the only arterivirus PLP2 structure described is that of equine arteritis virus (EAV), a distantly related virus. Here we describe the first crystal structure of the PRRSV PLP2 domain both in the presence and absence of its ubiquitin substrate, which reveals unique structural differences in this viral domain compared to PLP2 from EAV. To probe the role of PRRSV PLP2 deubiquitinating activity in host immune evasion, we selectively removed this activity from the domain by mutagenesis and found that the viral domain could no longer downregulate cellular interferon production. Interestingly, unlike EAV, and also unlike the situation for MERS-CoV, we found that recombinant PRRSV carrying PLP2 DUB-specific mutations faces significant selective pressure to revert to wild-type virus in MARC-145 cells, suggesting that the PLP2 DUB activity, which in PRRSV is present as three different versions of viral protein nsp2 expressed during infection, is critically important for PRRSV replication.

## Author summary

Within the pork farming industry, outbreaks of porcine reproductive and respiratory syndrome virus (PRRSV) are extremely costly, and significant effort has gone in to the design and production of vaccines aimed at limiting the impact of PRRSV outbreaks. To better understand the specific mechanisms used by PRRSV to suppress host immune responses during infection, we investigated the structure of a critical multifunctional PRRSV protease, PLP2, which processes the viral polyprotein during replication, and which is capable of suppressing host antiviral immune responses. Using a crystal structure of PLP2 bound to its cellular substrate ubiquitin (Ub), we characterized the residues forming the substrate binding surface. This was used as a platform to guide the design of PLP2 mutants with reduced capacity to bind ubiquitin, and provided a clear link between PLP2 deubiquitinating activity and host innate immune evasion. Using structure-guided mutagenesis and reverse genetics methods for PRRSV, we demonstrate that specific PLP2 residues that are critically involved in substrate recognition are under selective pressure to revert to wild-type during infection. These findings are in contrast to what has been observed with the equine arteritis virus (EAV) PLP2 domain, and uniquely highlight the specific importance of PRRSV PLP2 deubiquitinating activity with respect to viral replication.

## Introduction

The porcine reproductive and respiratory syndrome virus (PRRSV) is a positive-sense, single-stranded RNA virus and a member of the family *Arteriviridae*. The most pervasive infectious disease within swine industry [1], PRRSV outbreaks travel rapidly through swine herds causing reproductive failure and respiratory distress in infected pigs. Economic losses in the United States associated with PRRSV infection have been estimated at over $650 million annually, and thus considerable efforts have been invested towards the improvement of current generation vaccines, and the investigation of novel approaches towards PRRSV vaccine development. Two distinct PRRSV species within the genus are known to circulate and cause disease in livestock. The PRRSV Lelystad strain (former genotype-1, currently PRRSV-1) and PRRSV VR-2332 strain (former genotype-2, currently PRRSV-2) were isolated in the Netherlands and the USA, respectively, and share roughly 60% nucleotide identity [2]. Currently, a number of modified-live virus (MLV) vaccines are in use to manage PRRSV-1 and PRRSV-2 outbreaks in livestock [3], however most suffer from a number of drawbacks including limited cross-protection against heterologous PRRSV strains and even reversion of vaccine strains restoring virulence has been observed [4,5].

PRRSV replication is accomplished with the help of four virus-encoded proteases–one of which is the papain-like protease 2 (PLP2), located within nsp2, which processes the nsp2|3 cleavage site to release nsp2 from the rest of the viral polyprotein [6]. Early bioinformatic analysis suggested that the PLP2 domain of PRRSV and other related arteriviruses belonged to the ovarian tumour domain (OTU) family of cysteine proteases [7]. Subsequent work demonstrated that several arterivirus OTU domain proteases hydrolyze ubiquitin (Ub) thereby downregulating Ub-dependent cellular innate immune signaling pathways as means to evade host antiviral mechanisms [8–11]. In addition to the N-terminal PLP2 domain, nsp2 carries a hypervariable region (HVR), a transmembrane (TM) domain, and a cysteine-rich (C) domain. The presence of a slippery a sequence at the junction between HVR and TM domains has been demonstrated to produce two alternate products nsp2TF, and nsp2N via -2 and -1 programmed ribosomal frameshifts, respectively [12,13]. These PLP2-containing products, which carry either an alternate transmembrane domain (nsp2TF), or which lack a transmembrane domain altogether (nsp2N) localize to different regions within the cell during infection, and likely serve unique functions.

The involvement of PLP2 in both replicative polyprotein processing, and deubiquitination of cellular substrates complicates the independent study of these functions, as both activities are dependent on the same active site residues. Previously, we have selectively inhibited the DUB activity of the prototypical equine arterivirus (EAV) PLP2 domain, and the Middle East respiratory syndrome coronavirus (MERS-CoV) papain like protease domain (PL^pro^) [9,14]. To achieve this, crystallographic data defining the Ub binding interface of these viral enzymes was used to guide the introduction of site-directed mutations that perturb the binding interface and block Ub binding without affecting the enzymatic activity needed for polyprotein processing. Here we describe a similar approach to selectively reduce the deubiquitination capacity of the PRRSV PLP2 domain without removing its polyprotein processing function. To this end, we determined the crystal structure of the PRRSV PLP2 domain both in the presence and absence of its Ub substrate to a resolution of 2.85Å and 2.3Å, respectively. The structures reveal a unique core OTU-like protease domain comprised of a 4-helix bundle, that deviates significantly from the closest structural homologue, the EAV PLP2 domain [9]. Using both Ub-bound and unbound structures of the PRRSV PLP2 domain, we highlight structural rearrangements that take place during substrate binding, and characterize the PLP2:Ub interface and unique Ub binding orientation. Using this structure as a guide, we selectively disabled PLP2 DUB activity by site directed mutagenesis while preserving polyprotein cleavage activity, providing explicit evidence to support the role of PLP2 in downregulating cellular interferon-β (IFN-β) production, similar to its EAV counterpart. Finally, using a PRRSV reverse genetics system, we assessed the replication of PLP2 DUB mutants, simultaneously targeting the nsp2, nsp2TF and nsp2N viral gene products. Surprisingly, we found that the DUB activity of PRRSV PLP2 appears critical to the replication of the virus, which contrasts our previous results concerning the DUB activity of EAV PLP2.

## Results

### Crystal structure of the PRRSV PLP2 domain in its apo-form, and in complex with Ub

**Fig 1:**
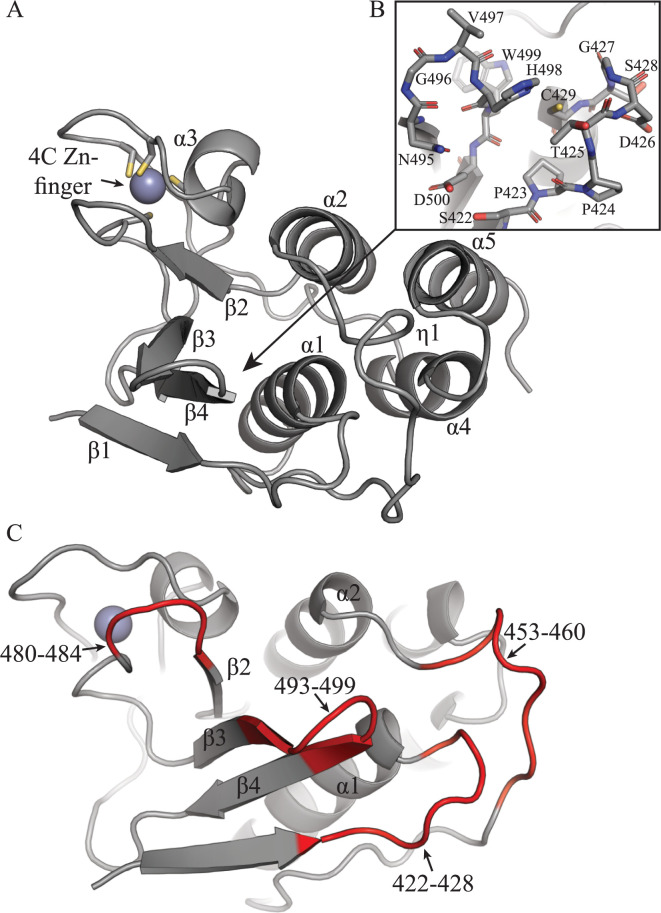
Crystal structure of the PRRSV SD01-08 PLP2 domain. (A) The PLP2 domain is shown as a cartoon diagram in gray, with secondary structure elements indicated. The α- and β-lobes are formed by α-helices 1, 2, 4 and 5, and β-strands 1–4, respectively. The 4-cysteine zinc finger is indicated. (B) The PLP2 active site is shown with active site residues Cys429 and His498 shown as sticks. (C) PLP2 domain with 422, 453, 480 and 493 loop structures highlighted in red.

To begin characterization of the PRRSV PLP2 domain structure, we determined the crystal structure of the enzyme from PRRSV Type I strain SD01-08 (Fig 1; Table 1) [15]. The SD01-08 PLP2 domain crystallized in space group C222_1_, with 2 copies present in the asymmetric unit. The 2 copies superposed with an RMSD of 0.635 Å over 144 C_α_ atoms, with unremarkable differences between the structures. Residues 385–415, representing the N-terminus of nsp2 following polyprotein cleavage were not visible in electron density maps.

**Table 1:** X-ray data collection and refinement statistics.

*Crystal*	SD01-08 PLP2	DV PLP2-Ub
*X-ray source*	CLS 08ID-1	Rigaku MicroMax-007HF
*Crystal geometry*		
Space group	C222_1_	C222_1_
Unit cell (Å)	a = 67.50 b = 102.35 c = 100.74; α = β = γ = 90°	a = 91.69 b = 159.49 c = 147.32; α = β = γ = 90°
*Crystallographic data*		
Wavelength (Å)	0.97934	1.45187
Resolution range (Å)	37.55–2.30 (8.91–2.30)*	45.84–2.85 (9.01–2.85)*
Total observations	64888 (6397)	117024 (16925)
Unique reflections	15677 (1516)	24728 (3640)
Multiplicity	4.1 (4.2)	4.7 (4.6)
Completeness (%)	99.1 (98.7)	97.1 (99.1)
*R*_merge_	0.042 (0.760)	0.169 (0.793)
CC1/2	0.998 (0.845)	0.987 (0.685)
I/σI	16.7 (2.1)	7.9 (2.0)
Wilson B-factor (Å^2^)	67.16	26.80
*Phasing statistics*		
FOM	0.32	
FOM after DM	0.60	
*Refinement statistics*		
Reflections in test set	1565	1156
Protein atoms	2308	5577
Zinc atoms	2	3
Solvent molecules	18	172
*R*_work_/*R*_free_	0.1999/0.2530	0.2148/2550
*RMSDs*		
Bond lengths/angles (Å/°)	0.010/1.48	0.009/1.28
*Ramachandran plot*		
Favored/allowed (%)	93.29/4.70	96.05/3.53
*Average B factor (Å* ^*2*^ *)*		
Macromolecules	60.64	45.32
Solvent	85.32	26.23

*Values in parentheses refer to the highest resolution shell

Overall, PLP2 adopts a similar, compact OTU-like fold similar to that of the previously determined EAV PLP2 domain [9], comprising of α and β lobes, as well as a structural 4-cysteine zinc finger (Fig 1A), and a DALI search [16] indicates the PRRSV PLP2 domain most closely resembles that of EAV PLP2. Interestingly however, PLP2 does display some notable deviations from the EAV PLP2 structure. In particular, the α-helical lobe of the PRRSV PLP2 is comprised of a 4-helix bundle as opposed to a 3-helix bundle seen in EAV PLP2. A sequence alignment of arterivirus PLP2 domains clearly indicate the absence of this additional helix (helix α5 of PRRSV-1 PLP2) in the EAV PLP2 domain (S1 Fig). Interestingly there is also limited sequence similarity between the SD01-08 helix α5 region and that region in the PLP2 domain of PRRSV VR2332, a prototypical type-II strain, and the PLP2 domain of the lactate dehydrogenase elevating virus (LDV) (S1 Fig). Additional structural work will be needed to determine whether the α-helical lobe of these latter proteases adopt a 3- or 4-helix bundle.

### Active site organization

Generally, the active site of cysteine proteases are comprised of a nucleophilic Cys, a His residue which polarizes the nearby Cys, and a third polar residue (generally Asp or Asn) proceeding the catalytic His, which coordinates and positions the His residue in an appropriate orientation for catalysis. The PRRSV PLP2 active site however is made up of a catalytic dyad formed by C429 and H498 and lacks an obvious third coordinating residue (Fig 1B). This type of active site arrangement is found in other OTU DUBs including A20, Cezanne, TRABID and the turnip yellow mosaic virus (TYMV) PRO domain [17–20]. Additionally, in the apo structure, H498 is positioned away from C429 in a catalytically incompetent conformation. Although unusual, similar active site conformations have been characterized in the OTU DUBs Cezanne, OTULIN, Gumby, OTUB1 and TYMV PRO [17,19,21–23].

### Ubiquitin binding to PLP2

**Fig 2:**
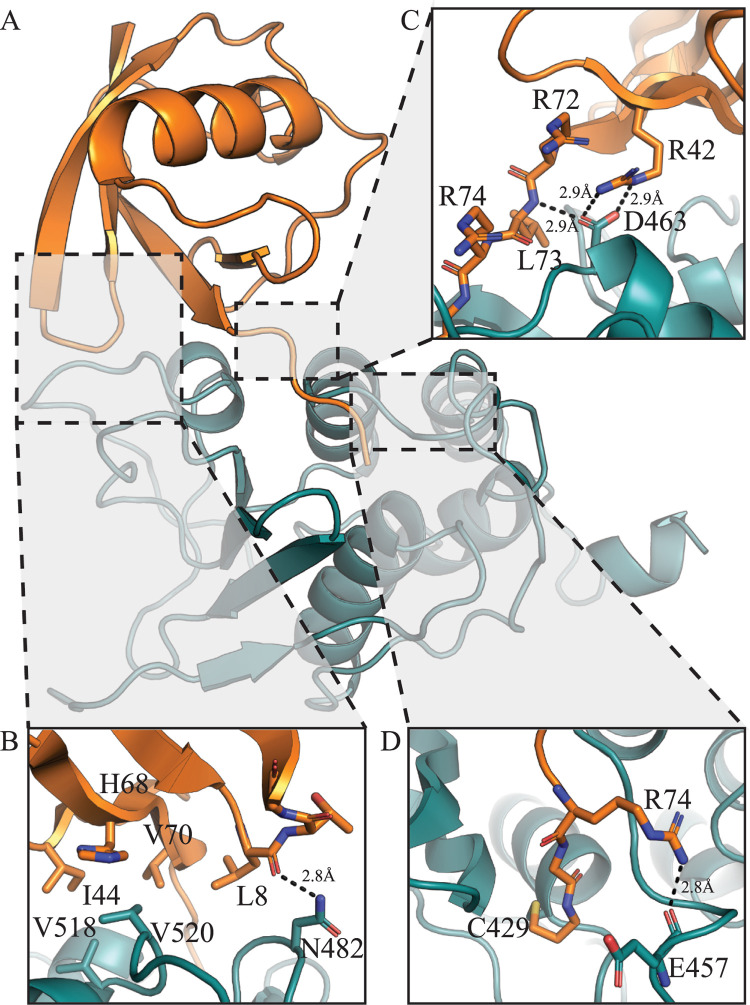
Crystal structure of the PLP2-Ub complex. (A) The PLP2 domain is shown in teal, with the bound Ub domain shown in orange. Interactions relevant with respect to Ub binding are shown in dashed boxes, with detailed interactions highlighted in panels B-D.

**Fig 3:**
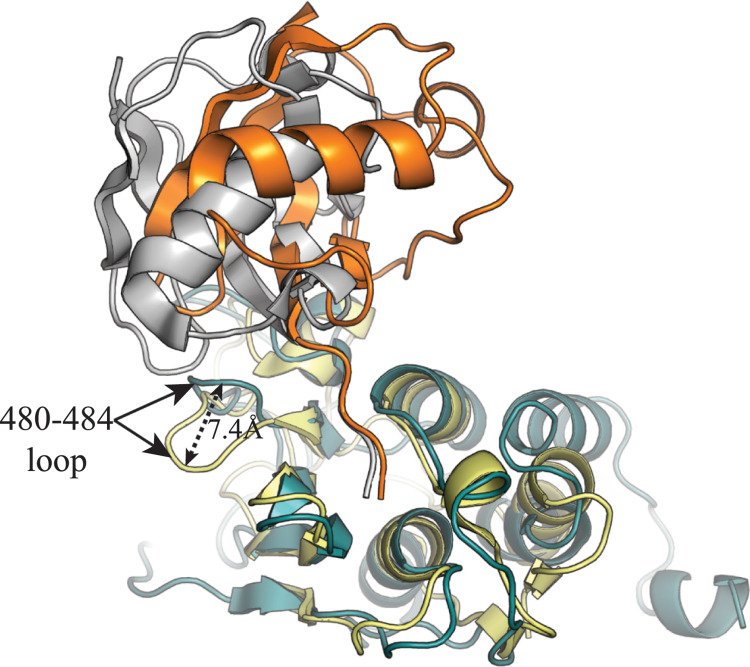
Comparison of the PRRSV and EAV PLP2 (4IUM) domains bound to Ub. The PRRSV and EAV PLP2 domains are depicted as teal and yellow cartoon diagrams, respectively, with Ub represented as orange and gray cartoons bound to the PRRSV and EAV PLP2 domains, respectively. Highlighted with arrows is the PRRSV 480–484 loop and homologous EAV PLP2 structure, with distance between the two structures indicated.

To determine the residues involved in Ub recognition by the PRRSV PLP2 domain, we were able to determine a crystal structure of the PLP2 domain of PRRSV strain DV bound to Ub (Fig 2). Attempts to determine a structure of Ub bound to PLP2 from PRRSV strain SD01-08 were unsuccessful, despite a 94% identity between the PLP2 domains of the two strains. Formation of the PLP2-Ub complex was facilitated by modifying the C-terminus of Ub with a reactive probe that forms a covalent adduct with the active site cystine of the PLP2 domain. The PLP2-Ub complex crystallized in space group C222_1_ with 3 copies of the complex in the asymmetric unit. The β-grasp fold of Ub interacts with the PRRSV PLP2 surface in a similar manner to that of EAV PLP2. Ub binding appears to be mediated primarily by V518 and V520, which interact with the hydrophobic I44 patch formed by Ub residues L8, I44, H68 and V70 (Fig 2B). Additional salt bridge interactions are found between PLP2 residue D463 and Ub residue R42 (Fig 2C), and a hydrogen bond between the backbone carbonyl of Ub residue L8 and the sidechain of PLP2 residue N482 (Fig 2B). Direct interactions between Ub, and a PLP2 loop structure comprised of residues 453–460 (453 loop; Fig 1C) are also observed, with Ub residue Arg74 forming an H-bond with the backbone carbonyl of PLP2 E457 (Fig 2D). A comparison of the PRRSV PLP2-Ub complex with the EAV PLP2-Ub complex shows that that Ub binds in a slightly different orientation, with the PRRSV PLP2-bound Ub tilted further towards the α-helical lobe of PLP2 (Fig 3). This shift in substrate orientation is mediated by a PLP2 loop formed by residues 480–484 (480 loop; Fig 1C), which is shifted roughly 7.4 Å toward the Ub-binding interface with respect to the homologous EAV PLP2 structure, and which would otherwise clash sterically with Ub (Fig 3).

**Fig 4:**
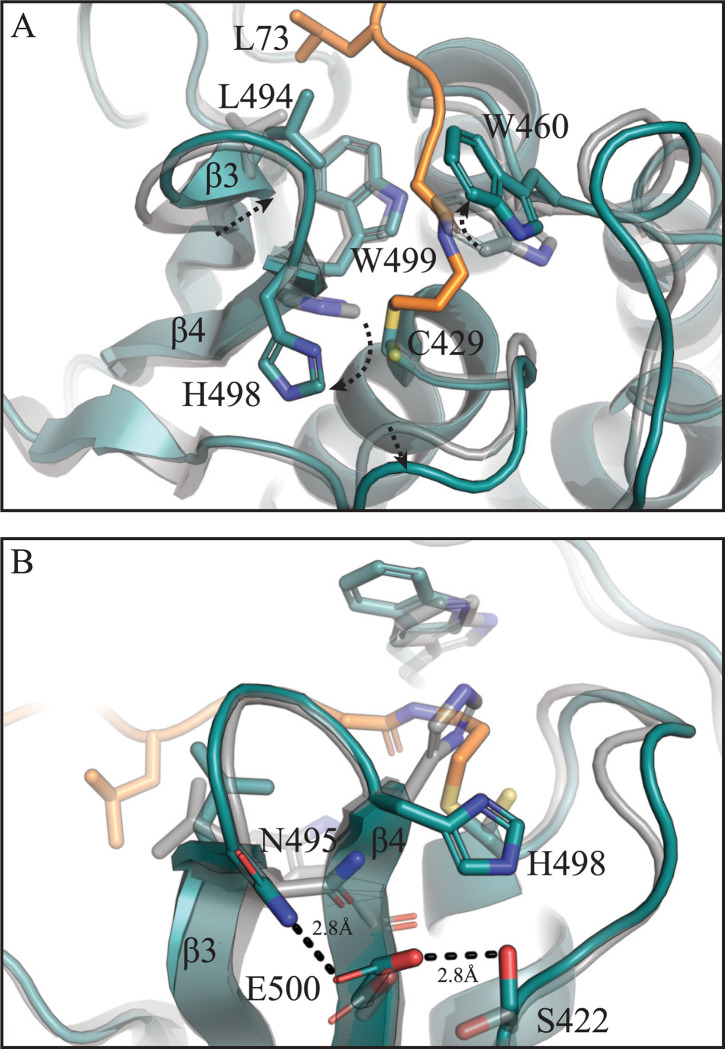
PLP2 active site movement upon Ub binding. (A) The apo PLP2 (transparent gray) and PLP2-Ub (teal) structures are overlaid to highlight PLP2 active site changes upon Ub binding. Dashed arrows indicate the direction of movement upon Ub binding, with Ub shown in orange. (B) Hydrogen bond network formed during Ub binding between β-strands 3 + 4, with hydrogen bonds shown as dashed lines.

A comparison of the apo and Ub-bound PRRSV PLP2 structures shows that Ub-binding results in a repositioning of H498 in a catalytically competent conformation, although there is no obvious direct coordination by nearby residues (Fig 4). The repositioning of H498 instead may be mediated by the stabilization of mobile structural elements within PLP2 that occurs upon Ub binding. Structural regions surrounding the active site of PRRSV PLP2 show a high degree of mobility, as evidenced by higher B-factors in these areas (S2 Fig). These regions include the β3-β4 loop (residues 493–499) which contains H498, the 453 loop which extends toward helix α2, an active site loop (residues 422–428) extending towards the catalytic C429, which also forms the oxyanion hole, and the 480 loop situated near the Ub-binding region (Fig 1C). Stabilization of the β3-β4 loop is mediated by hydrophobic interactions between W499 and L494 of PLP2 and L73 of Ub. These interactions move the loop inward toward the core of PLP2, and also permit the formation of an H-bond network between N495-E500-S422 (Fig 4), effectively linking the β3-β4 loop with the active site loop. This hydrogen bonding network also orients the 422–428 loop away from the active site, permitting H498 to swing into position. Importantly, all residues found to be involved in Ub binding are conserved between DV and SD01-08 strains (S3 Fig).

### Probing the deubiquitinating activity of PRRSV PLP2

**Fig 5:**
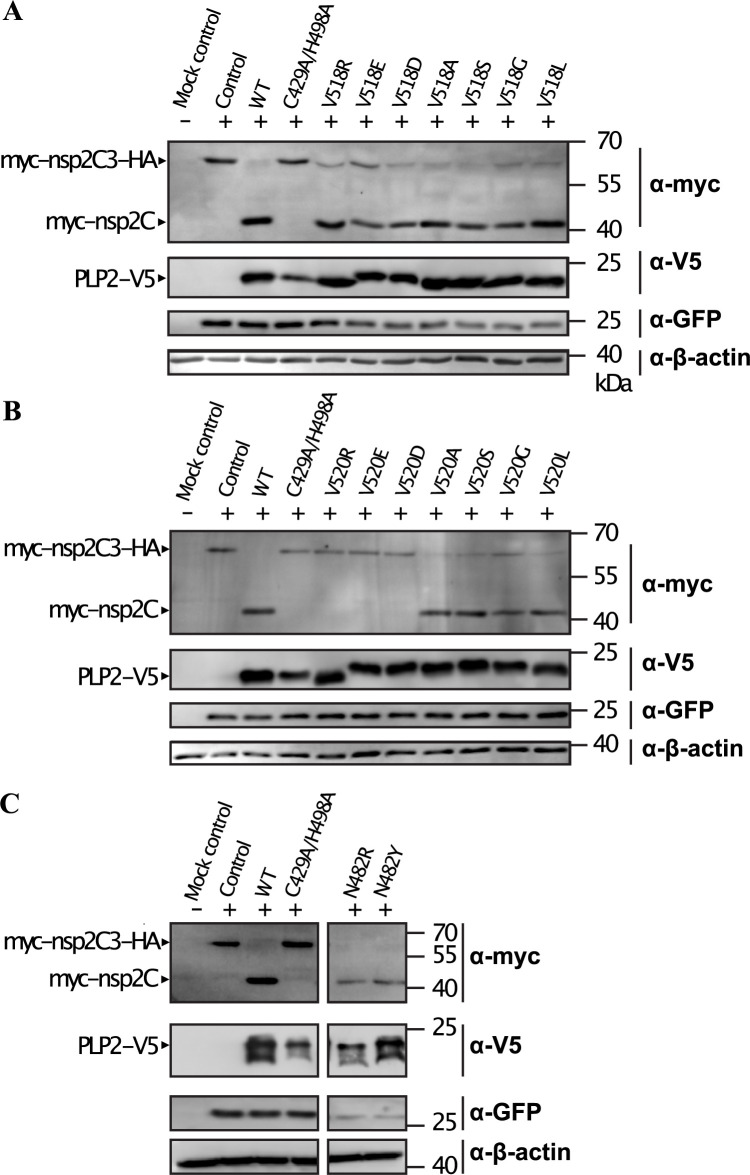
In trans nsp2C|3 protease activity of PLP2 mutants in vitro. HA-nsp2C|3 was co-transfected alongside wild-type PLP2, or a series of PLP2 mutants at position V518 (A), V520 (B) or N482 (C), and cleavage products were visualized by Western blot. Efficient trans cleavage is indicated by the appearance of the myc-nsp2C cleavage product mediated by PLP2. The active site PLP2 mutant C429A/H498A is included as a control. Membranes were probed with anti-GFP and anti-actin antibodies as transfection and loading controls, respectively.

The PLP2 deubiquitinating and polyprotein processing activities rely on the same active site residues to facilitate peptide bond hydrolysis. As PLP2-mediated polyprotein cleavage is necessary for PRRSV replication [24], this precludes the generation of a catalytic knockout as a means to study PLP2 DUB activity in the context of replicating PRRSV. To circumvent these limitations, we targeted PLP2 residues N482, V518 and V520 by mutagenesis with the aim of preserving polyprotein cleavage activity (which presumably relies on PLP2 residues closer to the active site) and disrupting Ub binding. These residues were found to be directly involved in Ub binding (Fig 2B), yet were distant from the enzyme active site. Mutations at these positions were therefore deemed unlikely to interfere with polyprotein processing. First, to verify that mutations did not inhibit PLP2 polyprotein cleavage activity, a *trans* cleavage assay was performed as previously described [9,14]. HEK293T cells were transfected with a construct encoding an nsp2-nsp3 fusion including the PLP2 recognition site and flanked by myc and HA epitope tags (myc-nsp2C-3-HA), and constructs encoding the soluble PLP2 domain and corresponding mutants. Polyprotein cleavage activity was monitored qualitatively as a shift in mass following PLP2-mediated proteolysis at the nsp2-3 cleavage site. Introduction of a long, negatively charged Glu at position 518 had the most significant effect on polyprotein processing activity (Fig 5), while Ala, Asp, Gly, Leu and Arg mutations had a minor effect, and Ser had little or no effect (Fig 5). With respect to position 520, Arg, Asp and Glu mutants abolished polyprotein activity entirely, while conservative Ser, Gly and Leu mutants were minorly affected, and the Ala mutant retained wild-type level cleavage activities (Fig 5). For position 482, the addition of large or charged residues (Arg, Tyr) had no effect on polyprotein processing (Fig 5). These results suggest that PLP2 residues at the Ub-binding interface can be targeted for mutagenesis without significantly interfering with polyprotein cleavage and presumably viral replication. Additionally, the observation that only conservative mutations at positions 518 and 520 were tolerated suggests that these residues may be involved in viral polyprotein processing, although it remains possible that less conservative mutations interfered with PLP2 folding and/or stability during expression.

**Fig 6:**
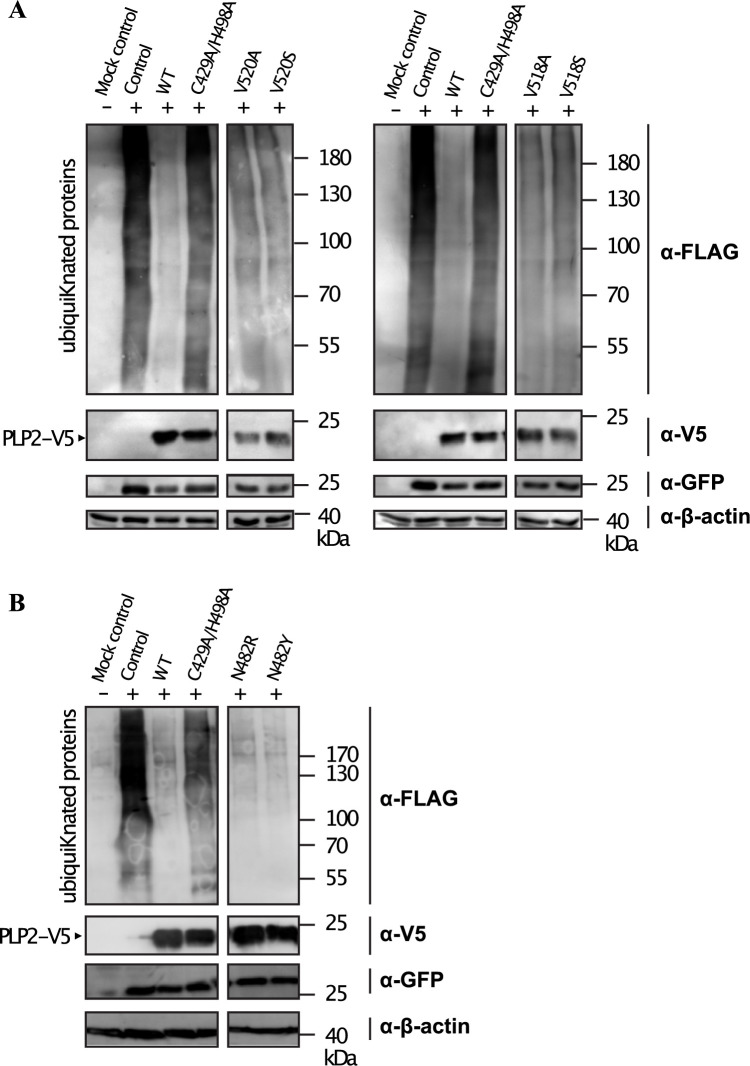
Global DUB activity of PLP2 mutants in vitro. The total amount of cellular FLAG-ubiquitinated proteins were visualized by Western blot using α-FLAG antibodies. Expression of FLAG-Ub in the absence of PLP2 resulted in a smear of FLAG-ubiquitinated proteins. In the presence of wildtype PLP2, total cellular FLAG-ubiquitinated proteins are significantly reduced. This activity is abrogated during co-expression of a PLP2 active site mutant (C429A/H498A). Rationally designed PLP2 mutants at position V520 and V518 (A) showed a moderate reduction in global DUB activity, while N482R and N482Y mutants were relatively unaffected (B).

To examine the effect of PLP2 mutants on global, cellular deubiquitinating activity HEK293T cells were co-transfected with FLAG-tagged Ub, and PLP2 mutants demonstrating little or no inhibition of polyprotein cleavage activity, as has been described previously [9,14]. As expected, co-transfection of wt PLP2 and Ub resulted in a marked decrease in cellular ubiquitinated proteins (Fig 6), and this activity was abrogated upon mutation of the PLP2 active site cysteine (Fig 6). The substitution of Ala or Ser at position 518 significantly reduced PLP2 DUB activity, as did substitution of Ala or Ser at position 520. In contrast, substitution of either Arg or Tyr at position 482 had no effect on PLP2 DUB activity. In agreement with the PLP2-Ub structure, residues V518 and V520 are critical with respect to Ub recognition, however residue N482 appears to be dispensable.

**Fig 7:**
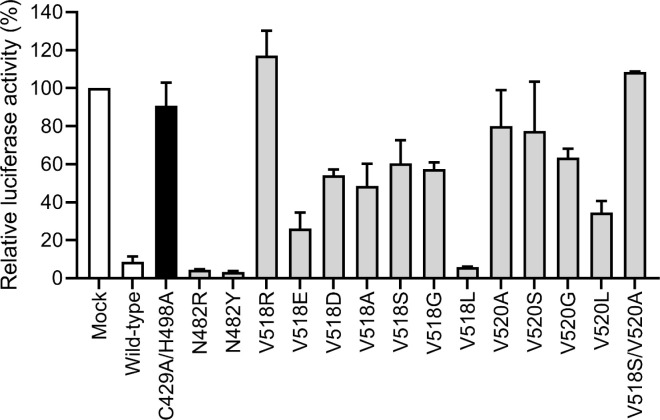
Inhibition of IFN-β promoter activity by rationally designed PLP2 mutants. HEK293T cells were co-transfected with plasmids expressing various PLP2 mutants, firefly luciferase under the control of the IFN-β promoter, renilla luciferase as endogenous control and RIG-I(2card) to induce the innate immune response. Wild-type PLP2 significantly inhibited IFN-β promoter activity, while the inactivated C429A restored this activity. Expression of DUB-specific V518 and V520 mutants all significantly increased IFN-β promoter activity with the exception of V518L, while both N482 mutants did not have an effect. All experiments were performed in triplicate and independently repeated at least twice. Bars correspond to the mean with error bars showing the standard deviation.

In response to the presence of cytoplasmic viral RNA, cellular pattern recognition receptors (PRRs) trigger the induction of Ub-dependent innate immune signaling cascades leading to the production and secretion of IFN-β, a hallmark antiviral cytokine. RIG-I in particular is one such PRR which has been shown to be directly deubiquitinated by arterivirus PLP2 domains, resulting in reduced levels of IFN- β expression [10]. To measure the effect of DUB-deficient PLP2 on cellular IFN- β production, we employed a dual luciferase-based reporter assay as has been previously described [9,14]. Briefly, firefly luciferase was placed under the control of the IFN-β promoter. Cellular innate immune signaling was induced via transfection of constitutively active retinoic acid-inducible gene I (RIG-I_(2CARD)_) [25] resulting in robust IFN- β promoter activity in the absence of PLP2. As expected, expression of wt PLP2 resulted in a significant reduction of RIG-I-dependent IFN-β promoter activity (Fig 7), and this activity was at least partly restored upon expression of PLP2 DUB mutants. Finally, combining DUB mutations to generate a PLP2 V518S/V520A double mutant restored IFN-β reporter activity to higher levels than observed with viruses carrying individual mutations (Fig 7). Together, these data suggest that PLP2 interfered directly with Ub-dependent pathways involved in the induction of cellular IFN-β, and that this activity is distinct from the polyprotein processing activities of PLP2.

**Fig 8:**
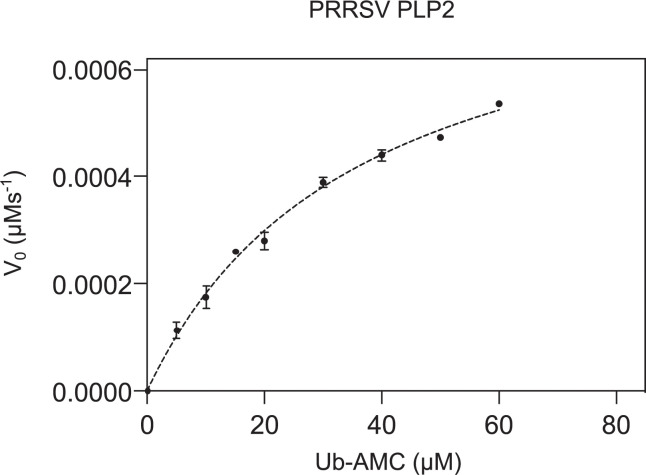
Michaelis-Menten plot of wt PRRSV DV_(385–578)_ PLP2. PLP2 (0.005 μM) activity was measured using Ub-AMC as substrate ranging from 0–60μM. Data points represent averages of at least three technical replicates, and error bars represent standard deviations.

Finally, the DUB activity of wt DV PLP2_(385–578)_ and its V518S, V520A, V518S/V520A and C429S/H498A mutants were measured *in vitro* using Ub-AMC as a fluorogenic substrate. Ub-AMC hydrolysis by wt PLP followed classical Michaelis-Menten behavior (Table 2, Fig 8). For the V518S, V520A, V518S/V520A mutants, specificity constants ($\frac{k_{cat}}{K_{m}}$) were determined due to an inability to achieve substrate saturation (Table 2). All mutations significantly reduce enzyme activity, with the V518S/V520A double mutant demonstrating the greatest effect and causing a loss of DUB activity that approached that of the catalytically compromised C429S/H498A mutant. In contrast to the Ub-AMC substrate however, the trans nsp2C|3 protease activities of the V518S, V520A mutations were not significantly altered compared to wt PLP2 (Fig 5). This confirms that these mutations have not significantly perturbed the enzyme active site, and that the relative losses in DUB activity observed in the PLP2 mutants in comparison to wt PLP2, are most likely caused by commensurate losses in Ub affinity as represented by *K_m_*, with the V520A mutation causing the greatest reduction in the enzyme’s affinity towards the Ub substrate.

**Table 2:** Michaelis-Menten kinetics parameters of PRRSV DV_385-578_ and mutants of PRRSV DV_385-578_ for Ub-AMC substrate.

Enzyme	K_cat_ (s^-1^)	K_m_ (μM)	K_cat_/K_m_ (μM^-1^ s^-1^)	% Relative activity^*a*^
**wt PLP2**	(1.7 ± 0.7) x 10^−1^	36 ± 6	(5 ± 2) x 10^−3^	100
**V518S**	ND^*b*^	ND	(1.1 ± 0.4) x 10^−3^	22 ± 9
**V520A**	ND	ND	(1.9 ± 0.2) x 10^−4^	4 ± 2
**V518S/V520A**	ND	ND	(1.33 ± 0.06) x 10^−4^	2.4 ± 0.9
**C429S/H498A**	ND	ND	(3.0 ± 0.2) x 10^−5^	0.6 ± 0.2

^*a*^Percentage of catalytic efficiency (K_cat_/K_m_) of enzymes relative to PRRSV DV_385-578_

^*b*^ND, not determined.

### Effect of DUB-specific PLP2 mutations on viral replication in vitro

**Fig 9:**
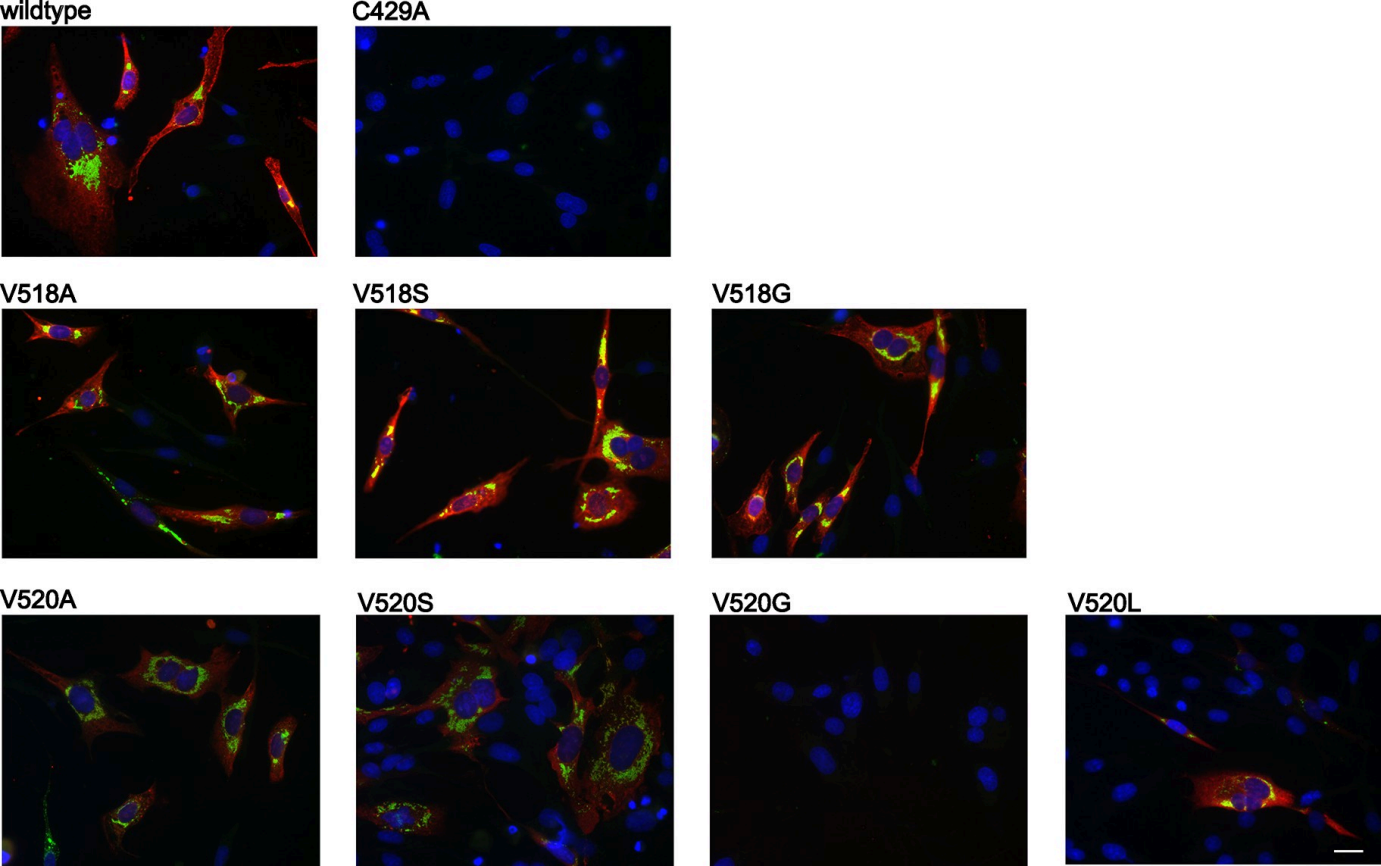
Immunofluorescence analysis of BHK-21 cells transfected with PRRSV DV. BHK-21 cells were transfected with mutant infectious RNA and infection was visualized using m-α-ORF7 together with d-α-m-Cy3 (red, showing the localization of the nucleocapsid protein N), and r-α-nsp2/3 (LV) together with g-α-r-Al488 (green). The nucleocapsid protein was found to localize throughout the cell in infected cells, and nsp2 was observed in the perinuclear region. Scale bar = 20 μm.

**Fig 10:**
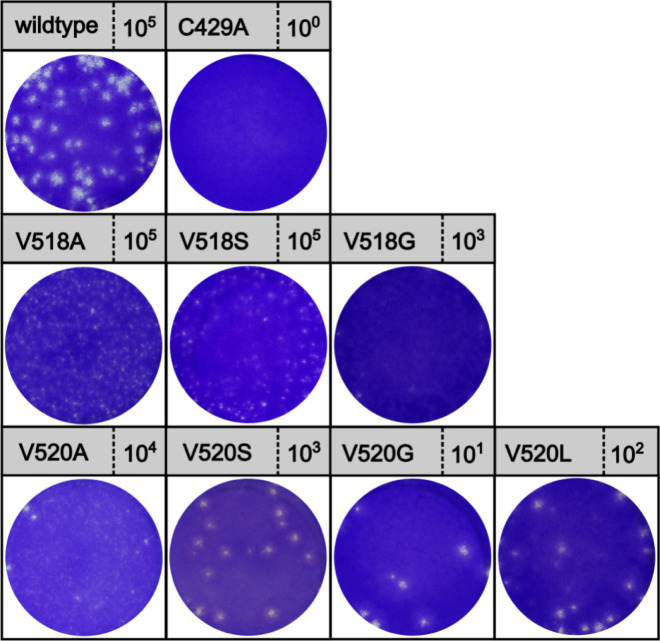
Plaque assay of BHK-21 harvest on MARC-145 cells. Virus-containing supernatant was harvested from transfected BHK-21 cells (p0) and used to infect MARC-145 cells. High titers in the range of 10^6^ to 10^7^ plaque forming units/ml were found for the wildtype virus, V518A and V518S mutants. The V518 mutants showed unique plaque phenotypes, appearing smaller and more diffuse that wild-type and V520 mutant plaques.

After confirming that several mutants at positions 518 (R, D, A, S and G) and 520 (A, S, G and L) retained polyprotein cleavage activity, showed lesser DUB activity and/or prevented inhibition of RIG-I-induced IFN-β promoter activity, each of these mutations were introduced into full-length PRRSV strain DV cDNA clones to observe their effect on viral replication. Immunofluorescence analysis of BHK-12 cells transfected with the mutated viral RNA confirmed the expression of viral ORF7-derived nucleoprotein (N) and nsp2, and therefore viral replication and production of structural proteins for V518 mutants (Fig 9). Nucleoprotein expression was lower for V520A/S/L mutants compared to V518 mutants, indicating either reduced replication or lower transfection efficiency for the V520 mutants. Interestingly, nucleoprotein could not be detected for the V520G mutant. Next, recovered virus was used to infect MARC-145 to confirm the production of infectious virus. Plaque assays performed on MARC-145 cells confirmed high viral titers for the V518A and V518S mutants, comparable to the wild-type virus titers, while lower titers were obtained for the V520 mutants (Fig 10). Notably, smaller, more diffuse plaque phenotypes were observed for the V518A, G and V518S mutants, which may indicate that the V518 mutants are more susceptible to cellular IFN or other innate responses in MARC-145 cells as a result of their reduced capacity to interfere with Ub-dependent pathways. The presence of plaques for the V520G mutant despite an inability to detect nucleoprotein expression can be attributed to the reduced sensitivity of the immunofluorescence assays.

To verify the genetic stability of recombinant PRRSV mutants, P1 virus was harvested following a single passage on MARC-145 cells. Sanger sequencing confirmed the stability of V518A and V518S mutations in the corresponding viruses after launch; however, the V520A and V520L mutants reverted to wild-type. Indeed, the V520S mutation was substituted to a cysteine residue after a single passage (while the indicator silent mutation remained intact), resulting in a loss of the intended serine mutation. Overall, only the recombinant PRRSV V518A and V518S mutants were stable after a single passage in MARC-145 cells. The high pressure to revert back to wild type suggests a significant inability to cope with the loss of DUB activity, which is markedly different from our experience working with other nidoviruses, such as the archetype arterivirus virus EAV and the Middle East Respiratory Coronavirus [9,14]. Overall, only the recombinant PRRSV V518A and V518S mutants were stable in MARC-145 cells although we have not assessed stability beyond the first passage.

## Discussion

Our data offers the first insight into the structure and substrate recognition of a PRRSV PLP2 domain. Unlike the prototypical EAV PLP2 domain [9], the core of the PRRSV-1 PLP2 domain is comprised of a 4-helix bundle which to date has evaded predictive bioinformatic tools. The PRRSV-1 domain active site undergoes significant rearrangement during Ub binding and adopts a catalytically incompetent conformation in the absence of Ub. PLP2 also interacts with Ub in a unique orientation compared to EAV PLP2, owing to the presence of an extended loop structure near the Ub binding site.

Previous studies have investigated PLP2 residues involved in DUB activity, as well as *cis* and *trans* polyprotein cleavage activity, albeit with the aid of homology models [26]. Notably, several residues were found to be critical for the DUB activity of the highly pathogenic HP-PRRSV strain JXwn06, and dispensable for *trans* polyprotein cleavage. Mutations at residue D85 (D459 according to our structure) inhibited PLP2 DUB activity, without affecting *trans* cleavage, however our structure shows no interaction of the D459 side chain with Ub (S4 Fig). The side chain of D459 does however support the 453 loop (S4 Fig) via interaction with the backbone amide of residue S551, and this loop structure does contain residues involved directly in Ub binding, namely the backbone carbonyl of residue E457, which forms a hydrogen bond with Ub residue R74 (Fig 2D). Alternatively, it is possible that this loop structure is involved in recognizing the proximal moiety of a diUb substrate bound across the PLP2 active site, thus acting as an S1’ Ub recognition site.

Additional mutations, including T88 (S462 in our structure) and D91 (D465 in our structure) were found to inhibit DUB activity when non-conservative, positively charged residues were substituted. Again, these residues are not directly involved in substrate binding as evidenced in our structures (S4 Fig) yet as postulated by the authors these mutations may inhibit DUB activity through electrostatic repulsion of the Ub substrate. Indeed, these residues are near the active-site-bound, positively charged C-terminus of Ub. Notably, mutations at positions T88, D85 and D91 which inhibited DUB activity were also found to specifically inhibit *cis* polyprotein cleavage activity in a *trans* cleavage-deficient PLP2 background, and failed to produce viable virus [26]. These residues are all located near the protease active site, and either directly or indirectly engage the linear C-terminus of Ub (S4 Fig). This suggests that common interactions are important during *cis* cleavage of the viral polyprotein, and that such interactions are less critical, or involve additional interactions with the viral polyprotein during *trans* cleavage. With respect to the mutations described here, our assays probed the effect on *trans* cleavage only, and further, investigated the structure of specific PLP2 residues in the context of experimentally determined PLP2 domains originating from PRRSV-1 strains. Further structural work will be necessary to determine how representative the PLP2 structures reported here are to those from HP-PRRSV and PRRSV-2 strains; to investigate the effect of these mutations on *cis* cleavage and to understand the structural requirements of PLP2 recognition of the viral polyprotein during replicative *cis* and *trans* cleavage activities.

Mutations at residue D89 (D463 according to our structure) were also previously found to inhibit DUB activity [26], *trans* nsp2-3 cleavage [6] and restores NFκB activation [27]. While homology models predicted that D463 may interact with the R72 side chain of Ub [26], our structure shows that this residue is actually involved in Ub recognition *via* a salt bridge interaction between D463 and Ub residue R42 (Fig 2C). Since R42 is distant from the conserved C-terminal _72_RLRGG_76_ sequence of Ub, it is difficult to predict how mutations at residue D463 influence polyprotein cleavage activity. Of note however, the D463 sidechain is also within hydrogen bonding distance of the backbone amide of Ub residue L73, which is found in the RLRGG motif of Ub. Since the extended structure of the Ub C-terminus forms several H-bond interactions with PLP2 residues lining the channel extending towards the active site, it’s plausible that similar interactions occur during *trans* cleavage of the nsp2|3 site. Mutation of D463 [27] would therefore disrupt engagement with Ub sidechain R42, and the backbone amide of the structurally equivalent residue to L73 at C-terminus of nsp2.

Residue 82 (456 in our structure) of HP-PRRSV and PRRSV-II PLP2 domains has been implicated in the specific cleavage of K63-linked polyUb chains [28], with interactions predicted to occur with a proximal Ub molecule at an S1’ binding site. Specifically, the presence of a serine at this position, as opposed to a proline was associated with increased activity towards K63-linked diUb substrates. In our structures position 456 is occupied by a proline, which is positioned on the 453 loop. As this loop structure was found to be involved in interactions with the bound monoUb, it’s possible that substitutions at this position influence binding of monoUb indirectly by altering the structure, or dynamics of this loop formation, in addition to potentially engaging proximal Ub molecules.

To date only a single arterivirus PLP2 domain has been described structurally [9], and no structural characterizations have been performed between substrate-bound and substrate-absent structures. This work provides the first structural insight into a PRRSV PLP2 domain and highlights active site rearrangements occurring during Ub substrate recognition. While these structures provide a detailed look at how monoubiquitin molecules are recognized by PRRSV-I PLP2, it remains to be determined if additional regions are responsible for engaging diUb molecules or larger polyUb chains. Future structural investigation using diUb substrates will hopefully shed light on these questions. Furthermore, several viral DUBs, including PRRSV PLP2, are capable of reversing conjugation of the antiviral ubiquitin-like modifier interferon stimulated gene 15 (ISG15) from cellular substrates. Interestingly, the 19 amino-acid stretch at the N-terminus of a PRRSV-1 PLP2 (residues 402–420) was found to be partially responsible for the inhibition of cellular IFN-β production, and for ISG15 processing in cell culture [11]. Our structures begin at residues 412 and 417 for the bound and un-bound structures, respectively, with the remaining N-terminal residues being disordered. Interestingly this N-terminal region is not in close proximity to the Ub-binding region of PLP2, suggesting that it is not involved directly in substrate recognition.

Using the structures that we have solved, we now identified critical residues responsible for PLP2 ubiquitin binding–most notably V518 and V520, which upon mutation would therefore likely disrupt the DUB function of PLP2. Indeed, several conservative substitutions at these positions reduced PLP2 DUB activity, while polyprotein cleavage remained intact, and they restored RIG-I-mediated IFN-β production using established reporter assays. Further, kinetic data measuring Ub turnover *in vitro* confirmed that the relative change in DUB activity for 518 and 520 mutants agreed with the change in IFN-β reporter activity, with mutations at position 520 having the most severe effect. This is also in agreement with immunofluorescence and plaque assay data, showing that more severely compromised DUB mutants have reduced expression of viral proteins, and produce lower viral titers.

These results show that in isolation, the DUB activity of PLP2 is separable from the polyprotein cleavage and is at least in-part responsible for downregulating host IFN production in the absence of additional viral proteins. DUB-specific mutation at positions V518 and V520 restored IFN-β promoter activity, and produced viable virus in a PRRSV reverse genetics system. While our observation that in some cases PRRSV mutations abrogating PLP2 DUB activity produced recombinant virus at titers comparable to wt PRRSV seems contradictory, it is important to note that the reporter assays used here are not expected to faithfully recapitulate the complexities of an actual viral infection, and do not take into consideration more subtle effects the mutations may have on the enzymatic activity, including differences in Ub chain linkage specificity, which may be differentially affected by specific PLP2 mutations, which may in turn lead to incongruous results when comparing to those obtained from assays measuring replication of complete virus. Further, the reporter assays and monoculture plaque methods used here are unable to place the effect of these mutations in the context of a PRRSV host with a functional innate and adaptive immune response, and do not measure the effect of PLP2 mutants on the expression of downstream interferon stimulated genes.

Surprisingly, in the case of V520 mutants, introduction of DUB-inhibiting mutations into recombinant PRRSV very quickly resulted in reversion to wild-type virus after passaging on MARC-145 cells, suggesting that unlike for EAV, PRRSV PLP2 DUB activity is critically involved in viral replication. The virus mutants with V518 substitutions were more stable in our hands, but showed a small plaque phenotype, suggesting that these viruses are also compromised. Further studies should characterize the stability of these viruses, and in what way the infection is influenced by these mutations. It may be that these mutants less efficiently disrupt the DUB function during infection after all, making their phenotype less severe, resulting in reduced selective pressure to revert to wild-type. The idea that PRRSV PLP2’s DUB function may have a more direct role in virus infection, besides having innate immune evasive activity, is supported by recent evidence showing that nsp2TF, the -2 ribosomal frameshift product of nsp2 which also contains the PLP2 protease [12] co-localizes with PRRSV membrane (M) and envelope glycoprotein (GP5) heterodimers. PLP2 in the TF product removes K48-linked Ub chains from these M and GP5 proteins, which prevents their proteosome-mediated turnover [29]. If M and GP5 are partially degraded when PLP2’s DUB function is alleviated as in our mutants, virus particles cannot be assembled efficiently, and therefore nsp2TF may be critical to infection, explaining why DUB KO mutants of PRRSV are crippled and restoration of the DUB function is prone to happen in order to rescue particle formation. Based on our previous experiences with the EAV PLP2 domain, our approach with PRRSV PLP2 was predicated on the assumption that a DUB-deficient recombinant virus would be fully replication competent and could therefore be used as an attenuated modified-live virus to address current challenges with existing PRRSV vaccines. Our evidence, however, shows that DUB KO mutations are poorly tolerated in PRRSV, suggesting that this strategy is unlikely to be effective *in vivo*. As discussed above, the fact that PRRSV expresses multiple versions of PLP2 through the nsp2TF products, which each may have particular functions that may play critical roles in the infection, makes mutation of the PLP2 sequence more impactful than in viruses that do not express these extra TF products such as EAV. Finally, it is worth noting that given the significant sequence diversity between PRRSV-1 and PRRSV-2 strains, further structural and biochemical studies will be needed to confirm whether the DUB activity of PRRSV-2 PLP2 is equally critical with respect to PRRSV replication.

A number of modified live virus vaccines are currently in use worldwide to mitigate potentially devastating and costly PRRSV outbreaks in industrial pig farming [3]. Despite these advances, PRRSV outbreaks remain a significant burden in commercial pig farming operations and merits the continued investigation of novel strategies to improve and optimize current generation vaccines. This work describing the first structure of a PRRSV PLP2 domain provides a platform to improving PRRSV vaccines through structure guided targeting of the PLP2 domain.

## Materials and methods

### Purification of the PRRSV SD01-08 and DV PLP2 domains

Residues 385–578, and 399–578 of the PRRSV SD01-08 and PRRSV DV polyproteins, respectively, were expressed off of vector pASK3 as previously described [9]. To purify the SD01-08 PLP2 domain, *E*. *coli* BL21 (DE3) Gold cells harbouring pASK-SD_385-578_ were grown to an OD_600_ = 0.8 under ampicillin selection and induced overnight at 16°C in the presence of 200 ng/mL anhydrotetracycline. The cell pellet from 1 L of culture was resuspended in lysis buffer containing 50 mM tris pH 7.2, 7.5 mM imidazole, 300 mM NaCl, EDTA-free Sigma*FAST* EDTA-free protease inhibitor, 5μg/mL DNase and 1mM tris(2-carboxyethyl)phosphine (TCEP). Cells were lysed via French press and lysate clarified at 17,200 x *g*. Lysate was incubated with Ni-NTA resin, and washed with 10CV lysis buffer followed by 20CV lysis buffer + 500mM NaCl. The resin was re-equilibrated in lysis buffer and eluted with the addition on 250 mM imidazole. Eluted protein was dialyzed overnight against 20 mM Tris pH 7.2, 250 mM NaCl, 3 mM DTT and purified by size exclusion chromatography (SEC) using a Superdex 75 (GE Healthcare) 16/60 column equilibrated in the same buffer.

To purify the PRRSV DV PLP2 domain, pASK-DV_399-578_ was transformed into *E*. *coli* C2523 (NEB) cells and induced under the same conditions described for the SD01-08 PLP2 domain. Cell pellets were resuspended in lysis buffer containing 50 mM Bis-Tris propane pH 8.5, 450 mM LiCl, 10% glycerol, 5 μg/mL DNase, 1 mM TCEP. Cells were lysed via French press incubated with Ni-NTA resin, and washed with 20CV lysis buffer, followed by 30CV wash buffer supplemented with 25 mM imidazole. The DV PLP2 domain was eluted with the addition of 500 mM imidazole.

### Purification of the Ub-3Br suicide substrate

Ub-3Br was prepared according to Borodovsky *et al*. [30], and dialyzed into 20 mM Tris pH 8.5, 450 mM LiCl, 10% glycerol prior to use. Following Ni-NTA purification, DV PLP2 was immediately coupled to Ub-3Br. Ub-3Br and DV PLP2 were incubated at a 2:1 molar ratio for 30 min at RT, and the resulting complex was dialyzed into screening buffer comprised of 20 mM Bis-Tris propane pH 8.5, 150 mM NaCl, 5 mM DTT and further purified using a 10/300 Superdex 75 SEC column equilibrated in screening buffer.

### Crystallization, data collection and structure determination

The SD01-08 PLP2 and DV PLP2-Ub complexes were concentrated to 4 mg/mL and 18.7 mg/mL, respectively, then reduced with the addition of a final concentration of 10 mM DTT immediately prior to crystallization. Initial crystallization screens were set up in sitting drop vapour diffusion format using 0.5μL + 0.5μL drops using a Crystal Gryphon robotics system (Art Robbins Instruments).

For the SD01-08 PLP2 structure initial hits were optimized in hanging-drop format at 20°C. For X-ray data collection, a crystal grown in 22% PEG 2000, 0.35M ammonium acetate, 0.1M HEPES 7.5 was swept through cryoprotectant containing crystallization solution supplemented with 20% glycerol and flash frozen in LN_2_. X-ray diffraction data was collected at the Canadian Light Source synchrotron on beamline 08ID-1. Images were integrated and scaled in XDS [31] and merged using Aimless [32] within the CCP4i2 software package. Initial phases for the SD01-08 PLP2 structure were determined by a single-wavelength anomalous diffraction (SAD) experiment. CRANK2 [33] was used for anomalous substructure identification, refinement and initial density modification. RESOLVE [34] within the phenix software package was used for further density modification of the initial experimental maps, and phenix.autobuild [35] was used to generate a starting model. Structure refinement and further model building was accomplished with phenix.refine [35] and Coot [36], respectively.

Crystals for the DV PLP2-Ub structure were harvested directly from initial screening plates incubated at 4°C grown in 20% PEG 3350, 0.2M Mg(NO_3_)_2_. A single crystal was flash frozen and X-ray diffraction data collected in-house on a Rigaku MicroMax 007-HF X-ray generator equipped with a Cu anode target and RAXIS IV++ imaging plate detector. X-ray diffraction images were integrated and scaled using XDS, and merged using Aimless. The data were phased by molecular replacement with MOLREP [37] using the SD01-08 PLP2 structure and ubiquitin (1UBQ) as search models. Subsequent refinement and model building was carried out in REFMAC and Coot, respectively. Structure factors and coordinates for the PRRSV PLP2 and PLP2-Ub complexes have been deposited to the PDB under PDB IDs 8EHN and 8EHO, respectively. All structural figures were generated using PyMOL [38].

### Cells and antibodies

HEK293T cells were cultured in Dulbecco’s modified Eagle’s medium (DMEM) supplemented with 10% (v/v) fetal calf serum (FCS, Bodinco DV), 100 U/ml penicillin, 100 mg/ml streptomycin, and 2 mM L-glutamine. BHK-21 cells were cultured in Glasgow’s minimal essential medium (GMEM, Gibco) supplemented with 8% (v/v) FCS, 10% (v/v) tryptose phosphate broth (TPB), 10 mM HEPES pH 7.4, 100 U/ml penicillin, and 100 mg/ml streptomycin. MARC-145 were cultured in DMEM supplemented with 8% (v/v) FCS, 100 U/ml penicillin, and 100 mg/ml streptomycin. DMEM and cell culture supplements were obtained from Lonza.

Primary antibodies that were used were mouse-α-β-actin (A5316, Sigma-Aldrich), rabbit-α-GFP [9], mouse-α-V5 (37–7500, Invitrogen), mouse-α-myc (9E10, Roche), mouse-α-HA (ab18181, Abcam), and mouse-α-FLAG (F1804, Sigma-Aldrich). As secondary antibodies, goat-α-mouse-HRP (P0447, Dako), swine-α-rabbit-HRP (P0217, Dako), goat-α-mouse-AlexaFluor488 (A11001, Life Technologies), and donkey-α-rabbit-Cy3 (711-165-152, Jackson). The antibody α-ORF7 (P3-05-A27, MSD Animal Health) was obtained using mouse antiserum. The antibody α-nsp2/3 LV was obtained using rabbit antiserum [13].

### DUB and trans cleavage assays

For *trans* cleavage assays, HEK293T cells were grown to 70% and transfected with a combination of pcDNA3.1 plasmids encoding GFP (transfection control; 0.25 μg), myc-nsp2C3-HA (0.25 μg), and the various PLP2-V5 constructs (0.25 μg). The total amount of DNA was kept constant at 2 μg by the addition of empty pcDNA3.1 vector. DUB assays were carried out using the same procedure, using a FLAG-Ub construct in place of myc-nsp2C3-ha. Transfections were performed using the calcium phosphate transfection method. At 18 hours post transfection (h.p.t.), cells were lysed in Laemmli sample buffer and heated for 10 minutes at 95°C. Protein samples were separated via SDS-PAGE and transferred to a PVDF membrane (Amersham Hybond P 0.2, GE Healthcare) using the Trans-Blot Turbo transfer system (Bio-Rad). Membranes were blocked overnight at 4°C in phosphate buffered saline with 0.05% Tween-20 (PBST) and 5% skimmed milk powder. Following antibody incubation the membranes were washed with PBST and developed using Pierce ECL Western blotting substrate (Thermo Scientific).

### Luciferase-based IFN-β promoter activity assay

HEK293T cells were grown to 60% confluence and transfected with a combination of expression constructs encoding an IFN-β inducible firefly luciferase (5 ng), constitutively expressed Renilla luciferase (5 ng), RIG-I_(2CARD)_ (25 ng) and various PLP2-V5 constructs (50 ng). The total amount of DNA was kept constant by the addition of an empty pcDNA3.1 vector. Transfections were performed as described before using the calcium phosphate transfection method. At 18 h.p.t., cells were lysed in passive lysis buffer (Promega) for 30 minutes. Cell debris was separated from the lysate by centrifugation cell lysate supernatant luminescence was measured with the dual-luciferase reporter assay system (Promega) using the EnVision Multilabel plate reader (Perkin Elmer). Firefly luciferase activity was normalized to *Renilla* luciferase and statistical significance was determined using an unpaired two-tailed Student’s *t*-test.

### Plasmids and reverse genetics

The PRRSV DV PLP2 gene (amino acid 386–578) was cloned into vector pcDNA3.1 (Invitrogen) with a C-terminal V5-tag. For reverse genetics experiments, PLP2 mutations were introduced in a pACNR1185 vector containing the PRRSV DV full-length cDNA clone. Substitutions were first generated in a pUC57 shuttle vector containing the first part of the PRRSV cDNA, base pairs 1 to 3416, using site-directed mutagenesis. An additional silent point mutation was introduced in the preceding codon, to be able to rule out contamination and/or reversion with/to the wildtype virus. The substituted cDNA part was exchanged with the wildtype sequence in the pACNR1185 vector by traditional cloning Methods.

The mammalian expression constructs for expression of PPRSV DV nsp2C-3 (amino acids 1065 to 1692) were cloned into a pcDNA3.1 vector (Invitrogen) and contained an N-terminal myc-tag and C-terminal HA-tag. Additional mammalian expression plasmids have been described elsewhere: pcDNA3.1 eGFP [10], pCMV FLAG-Ub [39], pLuc IFN-β [40], pRL-TK (encoding Renilla luciferase, Promega), and pEBG RIG-I_(2CARD)_ [25].

### RNA transfection and virus recovery

PLP2 mutations were introduced into a pACNR1185 vector containing the PRRSV DV full-length cDNA, as described above. The pACNR1185 vector was linearized using NotI (Roche) and purified by phenol-chloroform extraction. Linearized DNA was used for *in vitro* RNA transcription, using the Ampliscribe T7 high yield transcription kit (Epicentre). Viruses were launched using IFN-deficient BHK-21 cells by transfecting with 5 μg of the transcribed, purified RNA using the Amaxa cell line Nucleofector kit T in the Nucleofector 2b device (Lonza). Viruses were harvested 24 h.p.t. and stored at -80°C, or fixed in 3% PFA at 24 h.p.t. for immunofluorescence assays.

The virus titers in plaque forming units (pfu/ml) of the launched viruses and the plaque phenotype was determined by plaque assays on MARC-145 cells capable of IFN expression. At 1 h.p.i. the inoculum was replaced by an Avicel overlay (1.2% Avicel (FMC), 0.85 x DMEM, 100 U/ml penicillin, 100 mg/ml streptomycin, 42.5 mM HEPES pH 7.4 (Lonza), and 3% (v/v) FCS), and incubated for 5 days. Cells were fixed using 3.7% formaldehyde (Merck KGaA) in PBS and viable cells were colored using a crystal violet stain. The recovered viruses from BHK-21 cells were passaged on MARC-145 cells grown to 80% confluence. Cells were infected using a multiplicity of infection (m.o.i.) of 1 to 10 (0.5 ml of the virus stocks) and further cultivated in MARC-145 medium containing 2% (v/v) FCS. Viruses were harvested when CPE was observed, at 5 to 7 days post infection (d.p.i.). Mutant viruses were verified by Sanger sequencing using viral RNA was isolated from the harvested supernatants before and after passaging the virus on MARC-145 cells, using the QiAmp viral RNA kit (Qiagen). cDNA was prepared using the RevertAid H minus reverse transcriptase (Thermo Scientific) with random hexamers (Promega). A small amplicon covering PLP2 was amplified using the cDNA as a template with Pfu polymerase (Stratagene).

### Immunofluorescence microscopy

Cells were permeabilized using 0.2% Triton-X100 (Sigma) in PBS and viral proteins were labelled using 1:15,000 m-α-ORF7 together with 1:1000 r-α-nsp2/3 (LV) as the primary antibodies and 1:300 g-α-r-Al488 together with 1:1000 d-α-m-Cy3 as the secondary antibodies, in PBS with 5% bovine serum albumin (BSA, Sigma). Hoechst stain (Thermo Scientific) was used to visualize cell nuclei. Cells were visualized using a Zeiss Axioskop 2 fluorescence microscope with an Axiocam HRc camera and the Zeiss Axiovision (v4.8.2.0) software.

### Kinetic characterization of wild type and mutant PRRSV DV PLP2_385-578_

Site directed mutants of PRRSV DV PLP2_(385–578)_ were constructed using the Q5 site-directed mutagenesis kit E0554S from New England Biolabs. *E*.*coli* C2523 was co-transformed with mutants or wild type PLP2 and plasmid pCGI expressing Ubp1, as previously described [9]. Purification of PRRSV DV PLP2_(385–578)_ was performed in a manner similar to the SD01-08 PLP2_(385–578)_ domain used for crystallographic studies. Enzyme kinetics assays were performed at 25°C in black-round bottom 384-well microtiter plates (Corning Inc.) using Ub-AMC (R&D Systems) as substrate. Reaction progress was monitored by fluorescence using SpectraMax iD5 Multi-Mode Microplate Reader (Molecular Devices), and data were collected using SoftMax Pro Software. Data were analyzed using Sigmaplot and GraphPad 9.0. The deubiquitinase activity assay buffer contained 50mM Tris HCl pH 7.2, 100mM NaCl and 5mM DTT. Assays were run at the following enzyme concentrations: wild-type PLP2 (0.005μM), PLP2_V518S_ (0.5μM), PLP2_V520A_ (1.0μM), PLP2_V518S/V520A_ (1.0μM) and PLP2_C429S/H498A_ (1.0μM), and Ub-AMC concentrations ranging from 0–60 μM, 0–15μM and 0–10μM respectively for 3,600 s. Fluorescence was monitored at λ_Ex_/λ_Em_ = 360/360nm. Initial rates were calculated from the fluorescence traces using the equation d[AMC]dt=(dFdt)(dFd[AMC]) in which $\frac{dF}{dt}$ is the initial rate of fluorescence change (V_0_) and $\frac{dF}{d[AMC]}$ is the slope of the AMC standard curve. The initial rates for wild-type PLP2 were fit to the Michaelis-Menten equation ${\left(\frac{d[AMC]}{dt}\right)}_{0}=\frac{k_{cat}[enzyme][Ub-AMC]}{K_{m}+[Ub-AMC]}$ to obtain *k_cat_* and *K_m_* values; whereas, due to reduced activity, mutant forms of the enzyme could only be reliably fit to the linear portion of the Michaelis-Menten equation ${\left(\frac{d[AMC]}{dt}\right)}_{0}=\frac{k_{cat}[enzyme][Ub-AMC]}{K_{m}}$ (when [S] is much smaller than *K_m_*) to yield only specificity constants $\frac{k_{cat}}{K_{m}}$.

## Supporting information

S1 Fig Multiple sequence alignment of arterivirus PLP2 domains. Secondary structures as determined by the X-ray crystal structures of the PRRSV and EAV PLP2 domains are displayed at the top and bottom of the sequence alignments, respectively. Virus abbreviations: PRRSV, porcine reproductive and respiratory syndrome virus; EAV, equine arteritis virus; LDV, lactate dehydrogenase-elevating virus. GenBank accession numbers: PRRSV SD01-08, DQ489311.1; PRRSV VR2332, EF536003.1; EAV, DQ846750.1; LDV, U15146.1. Sequence alignment was performed using Probcons [41]. Figure generated using the ENDscript 2 webserver [42].
(PNG)

S2 Fig B-factor putty representation of the (A) PLP2 and (B) PLP2-Ub complex. Regions of the structure represented with thicker diameter tubes, and lighter green-yellow colouring indicate a higher degree of thermal motion. The surface loops stabilized upon Ub-binding are indicated with arrows.
(PNG)

S3 Fig Alignment of PRRSV SD01-08 (accession and DV PLP2 domains (sequences retrieved from GenBank accession numbers DQ489311.1 and MW674755.1 respectively). Sequence alignment was performed in JalView [43]. Conserved residues are highlighted in purple.
(PNG)

S4 Fig Structural detail surrounding the active site of the Ub-bound PLP2 domain. Residues previously implicated with PLP2 DUB activity, including D459, S462, D463 and D465 are labelled and shown as sticks (Ub residues numbers are italicized). PLP2 and Ub structures are shown in teal and orange, respectively. H-bonds are represented as dashed lines.
(PNG)
