# Supplementary figures and images for: Demonstrating the importance of porcine reproductive and respiratory syndrome virus papain-like protease 2 deubiquitinating activity in viral replication by structure-guided mutagenesis
Source: PLoS Pathog. 2023 Dec 14;19(12):e1011872. doi: 10.1371/journal.ppat.1011872 (PMC10754444; doi:10.1371/journal.ppat.1011872)

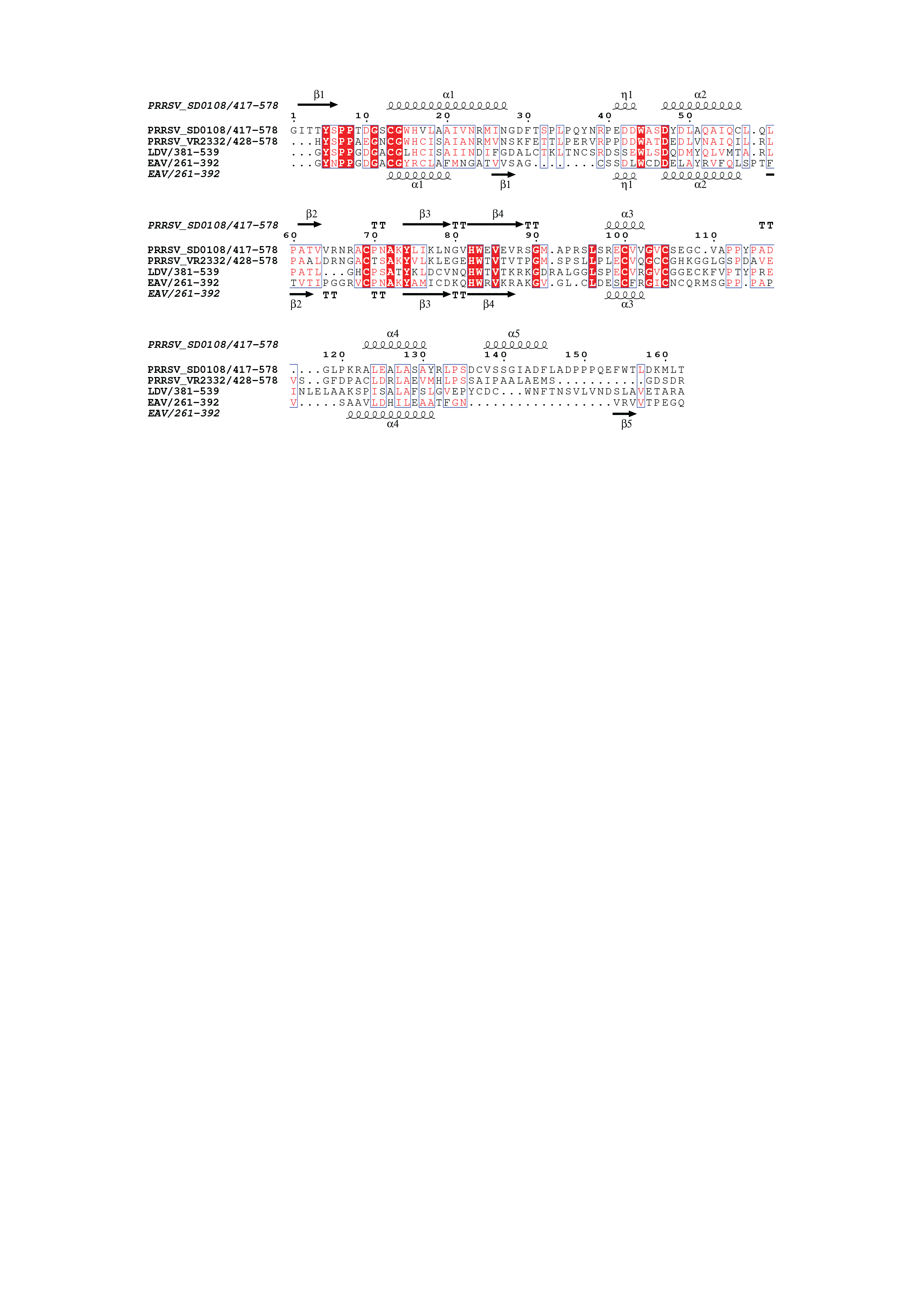

Supplement: S1 Fig — Secondary structures as determined by the X-ray crystal structures of the PRRSV and EAV PLP2 domains are displayed at the top and bottom of the sequence alignments, respectively. Virus abbreviations: PRRSV, porcine reproductive and respiratory syndrome virus; EAV, equine arteritis virus; LDV, lactate dehydrogenase-elevating virus. GenBank accession numbers: PRRSV SD01-08, DQ489311.1; PRRSV VR2332, EF536003.1; EAV, DQ846750.1; LDV, U15146.1. Sequence alignment was performed using Probcons [41]. Figure generated using the ENDscript 2 webserver [42]. (PNG) [file ppat.1011872.s001.png]

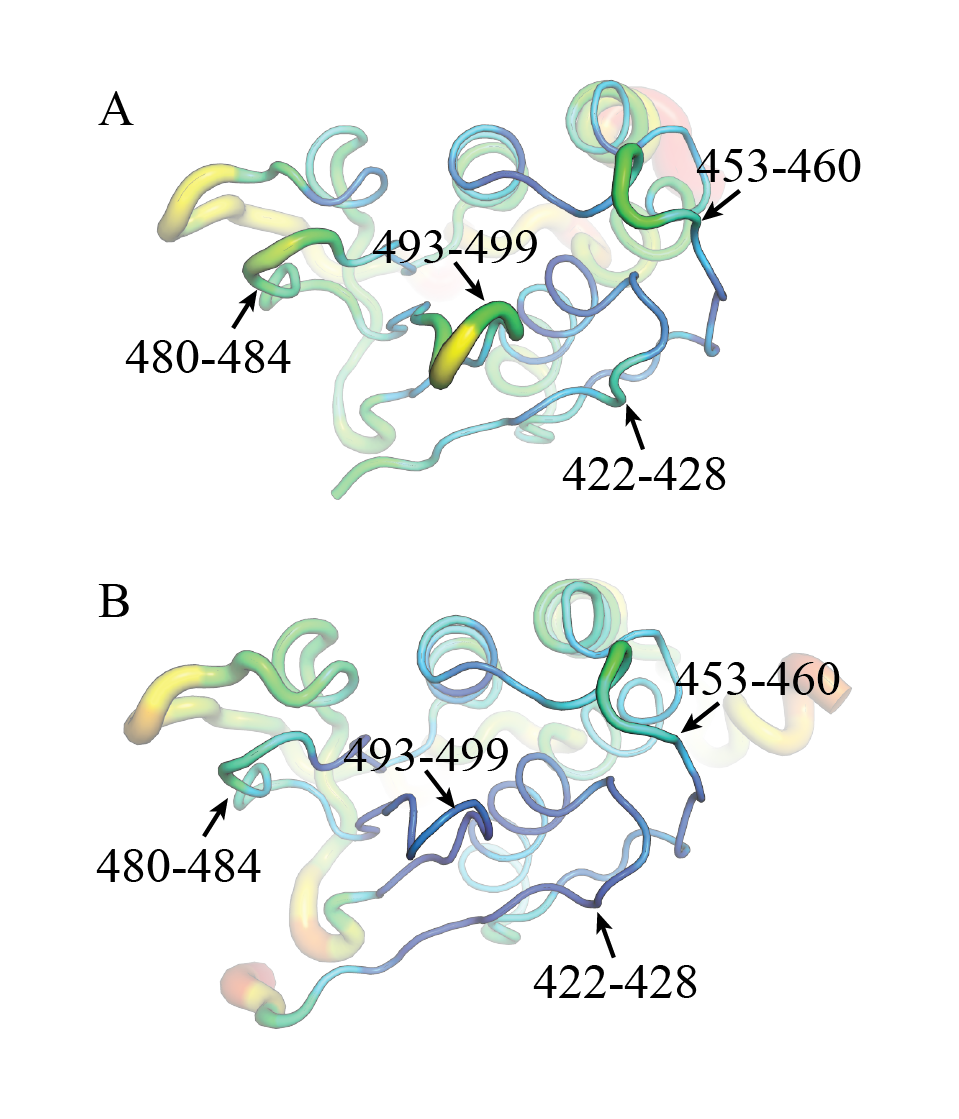

Supplement: S2 Fig — Regions of the structure represented with thicker diameter tubes, and lighter green-yellow colouring indicate a higher degree of thermal motion. The surface loops stabilized upon Ub-binding are indicated with arrows. (PNG) [file ppat.1011872.s002.png]

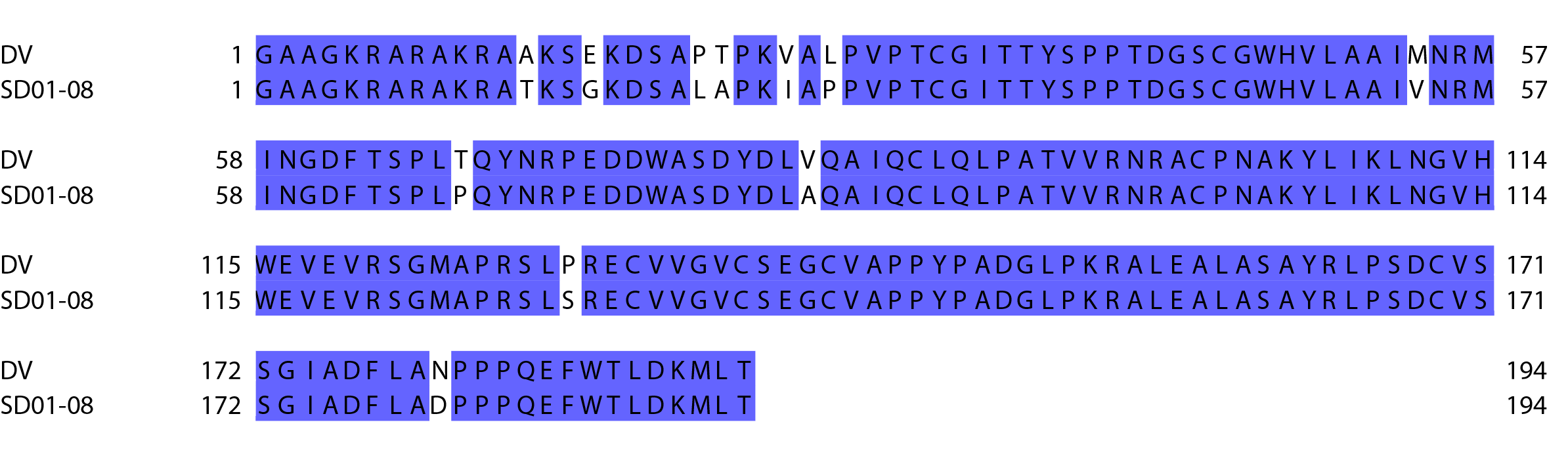

Supplement: S3 Fig — Sequence alignment was performed in JalView [43]. Conserved residues are highlighted in purple. (PNG) [file ppat.1011872.s003.png]

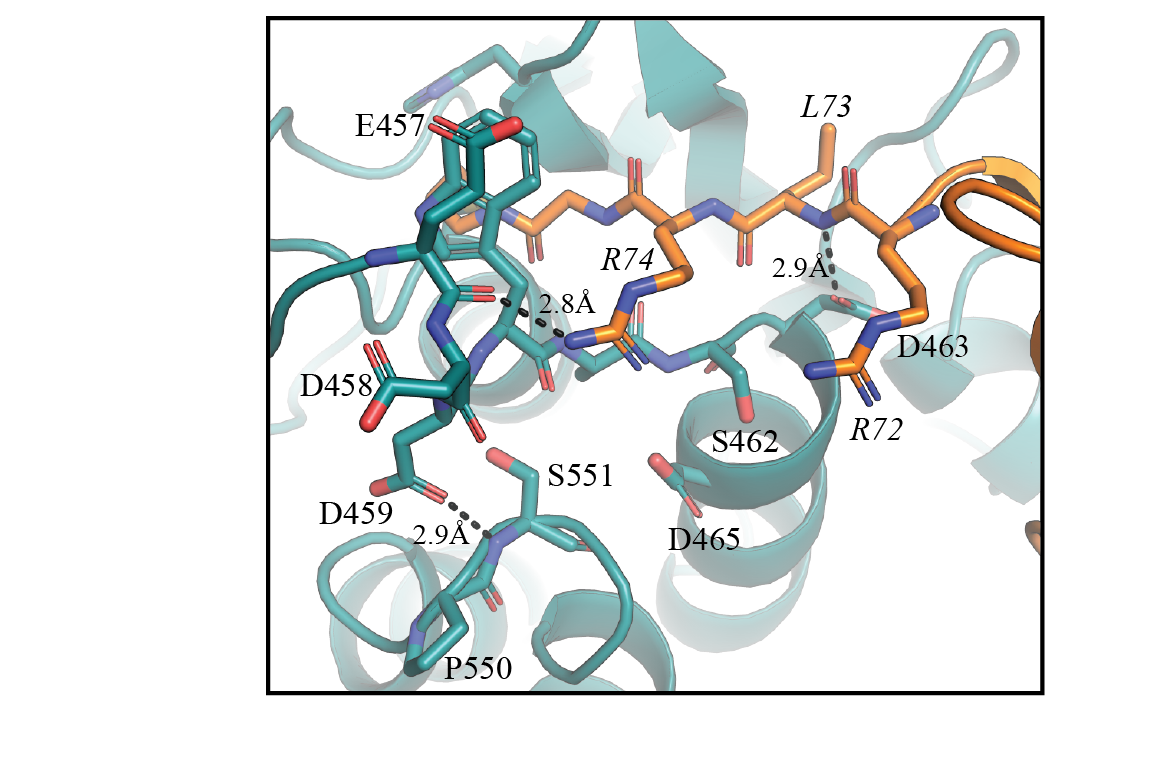

Supplement: S4 Fig — Residues previously implicated with PLP2 DUB activity, including D459, S462, D463 and D465 are labelled and shown as sticks (Ub residues numbers are italicized). PLP2 and Ub structures are shown in teal and orange, respectively. H-bonds are represented as dashed lines. (PNG) [file ppat.1011872.s004.png]
